# Inhibition of anlotinib-induced autophagy attenuates invasion and migration by regulating epithelial-mesenchymal transition and cytoskeletal rearrangement through ATG5 in human osteosarcoma cells

**DOI:** 10.1590/1414-431X2023e13152

**Published:** 2024-02-19

**Authors:** Bingxin Zheng, Xiangchen Sun, Li Zhang, Guojian Qu, Chongmin Ren, Peng Yan, Chuanli Zhou, Bin Yue

**Affiliations:** 1 Department of Orthopedic Oncology, The Affiliated Hospital of Qingdao University, Qingdao, China; 2 Department of Operating Room, The Affiliated Hospital of Qingdao University, Qingdao, China; 3 Department of General Surgery (adult), Qingdao Women and Children's Hospital, Qingdao, China; 4 Department of Spinal Surgery, The Affiliated Hospital of Qingdao University, Qingdao, China

**Keywords:** Anlotinib, Autophagy, ATG5, Metastasis, Osteosarcoma

## Abstract

The cure rates for osteosarcoma have remained unchanged in the past three decades, especially for patients with pulmonary metastasis. Thus, a new and effective treatment for metastatic osteosarcoma is urgently needed. Anlotinib has been reported to have antitumor effects on advanced osteosarcoma. However, both the effect of anlotinib on autophagy in osteosarcoma and the mechanism of anlotinib-mediated autophagy in pulmonary metastasis are unclear. The effect of anlotinib treatment on the metastasis of osteosarcoma was investigated by transwell assays, wound healing assays, and animal experiments. Related proteins were detected by western blotting after anlotinib treatment, ATG5 silencing, or ATG5 overexpression. Immunofluorescence staining and transmission electron microscopy were used to detect alterations in autophagy and the cytoskeleton. Anlotinib inhibited the migration and invasion of osteosarcoma cells but promoted autophagy and increased ATG5 expression. Furthermore, the decreases in invasion and migration induced by anlotinib treatment were enhanced by ATG5 silencing. In addition, Y-27632 inhibited cytoskeletal rearrangement, which was rescued by ATG5 overexpression. ATG5 overexpression enhanced epithelial-mesenchymal transition (EMT). Mechanistically, anlotinib-induced autophagy promoted migration and invasion by activating EMT and cytoskeletal rearrangement through ATG5 both *in vitro* and *in vivo*. Our results demonstrated that anlotinib can induce protective autophagy in osteosarcoma cells and that inhibition of anlotinib-induced autophagy enhanced the inhibitory effects of anlotinib on osteosarcoma metastasis. Thus, the therapeutic effect of anlotinib treatment can be improved by combination treatment with autophagy inhibitors, which provides a new direction for the treatment of metastatic osteosarcoma.

## Introduction

Osteosarcoma is the most common primary malignant bone tumor occurring in children and adolescents, has high metastatic potential, and is closely related to poor prognosis (1). Neoadjuvant chemotherapy combined with surgery is the current standard treatment approach for osteosarcoma, and the five-year survival rate has increased to approximately 70% (2). However, for osteosarcoma patients diagnosed with pulmonary metastases, the five-year survival rate is more than twofold lower. Almost all patients with recurrence have pulmonary metastases (3). Moreover, patients with lung metastases tend to exhibit chemoresistance, and their prognosis remains unsatisfactory.

The multitargeted receptor tyrosine kinase inhibitor anlotinib has been reported to have antitumor effects on soft tissue sarcoma and has an important role in the treatment of patients with advanced osteosarcoma. Thus, determining how to increase the efficacy and effective duration of anlotinib treatment is of great importance to improve the prognosis of patients with metastatic osteosarcoma. Autophagy plays a dual role in tumor metastasis, which may greatly affect the utility of anlotinib treatment for osteosarcoma. However, few studies have been reported on the antitumor mechanism of anlotinib in osteosarcoma. To the best of our knowledge, there are no studies reporting the effect of anlotinib treatment on autophagy in osteosarcoma, and there are no studies on the role of autophagy mediated by anlotinib in the metastasis of osteosarcoma.

In this study, the effect of anlotinib on osteosarcoma cell migration, invasion, and autophagy was investigated *in vitro* and *in vivo*. Additionally, we explored the role and molecular mechanism of autophagy mediated by anlotinib in the pulmonary metastasis of osteosarcoma.

## Material and Methods

### Cell lines and reagents

The human osteosarcoma cell lines 143B and KHOS were purchased from the American Type Culture Collection (ATCC). 143B and KHOS cells were cultured in DMEM (YC-2006, Nexell, China) and RPMI-1640 medium (YC-1002), respectively. The medium was supplemented with 10% fetal bovine serum (FBS) (Gibco, USA), 0.1 mg/mL streptomycin, and 100 U/mL penicillin (Solarbio, China). All cell lines were maintained at 37°C in 5% CO_2_.

The antibodies and materials used in the experiments were as follows: anti-rabbit P62 antibody (18420-1-AP; ProteinTech, China), anti-rabbit ATG5 antibody (10181-2-AP; ProteinTech), anti-rabbit LC3A/B antibody (12741; Cell Signaling Technology, USA), anti-rabbit LC3B antibody (3868; Cell Signaling Technology), anti-rabbit MMP-9 antibody (13667; Cell Signaling Technology), anti-rabbit N-cadherin antibody (ab76011; Abcam, UK), anti-rabbit E-cadherin antibody (208704-1-AP; ProteinTech), anti-rabbit Vimentin antibody (bs-0756R; Bioss, China), RhoA activation assay kit (BK036-S; Cytoskeleton, USA), anti-rabbit LIMK2 antibody (12350-1-AP; ProteinTech), anti-rabbit Cofilin antibody (10960-1-AP; ProteinTech), anti-rabbit LIMK2 (phospho T505) antibody (ab38499; Abcam), anti-rabbit Cofilin (phospho S3) antibody (ab12866; Abcam), anti-rabbit GAPDH antibody (AB0037; ShareBio, China), anti-rabbit IgG (Hazel) labeled antibody (AB0101; ShareBio). Anlotinib and Y-27632 2HCl were purchased from Selleck (USA) and were diluted to the desired concentration in the corresponding medium for follow-up experiments.

### RNA interference and ectopic expression

For gene knockdown, the human osteosarcoma cell lines 143B and KHOS were transfected with ATG5 shRNA (RiboBio, China) according to the manufacturer's instructions. After 24 h of incubation, the cells were treated with anlotinib and then cultured for another 24 h for follow-up experiments. The ATG5 shRNA sequences were as follows: #1, 5′-TTTCATTCAGAAGCTGTTT-3′ and #2, 5′-TTTCATTCAGAAGCTGTTT-3′. Cell lines with stable shATG5 expression were obtained by selection with 2 mg/mL puromycin (Solarbio).

For ectopic expression, the plasmid containing ATG5 cDNA or the negative control plasmid (RiboBio) was transfected into cells according to the manufacturer's instructions. After 24 h of incubation, the medium was replaced, and then the cells were treated with Y-27632 2HCl (10 µM) for 24 h.

### Cell viability assay

A Cell Counting Kit-8 (CCK-8; Dojindo, Japan) was used to estimate cell viability as per the protocols of the manufacturer. Briefly, 3000 cells were seeded into each well of 96-well plates, and the cells were treated with anlotinib at various concentrations. Then, viability analysis was carried out with CCK-8 reagent.

### Transwell assays

For the migration and invasion assays, after different treatments, 143B and KHOS cells were plated in chambers containing membranes coated with Matrigel (354480; Corning, USA) and membranes without a Matrigel coating (3422; Corning), respectively. The cells were fixed with methanol and then stained with Giemsa staining solution. Migrated cells were counted in five random fields per well under a microscope (Olympus, Japan).

### Wound healing assays

143B and KHOS cells were seeded into 6-well plates and cultured to 90% confluence. Then, a scratch was made carefully across the cell layer in each well using a 200 µL sterile pipette tip. Images of the wound were acquired after incubation for 0 and 24 h and analyzed using ImageJ (USA).

### Bioinformatics analyses

The potential interactions among VEGFR2, ATG5, and RHoA were investigated by bioinformatics analysis. Briefly, the target genes were manually entered into the website (http://genemania.org/), and the corresponding retrieval settings were used to examine the potential associations among these genes.

### Western blotting and GTPase activity assay

In brief, whole cell lysates were prepared using RIPA lysis buffer with protease inhibitors according to the manufacturer's instructions (Solarbio). Proteins were separated on 7.5-15% SDS-PAGE gels and then transferred to polyvinylidene difluoride (PVDF) membranes. After being blocked for 1 h, the membranes were incubated with the appropriate primary antibodies overnight at 4°C. The protein bands were ultimately visualized using enhanced chemiluminescence. The GTPase activity assay was performed according to the manufacturer's instructions (Cytoskeleton).

### Transmission electron microscopy (TEM)

Cells were digested with 0.25% trypsin and then fixed with 1.5% glutaraldehyde for 6 h at 4°C. The ultrathin sections (100 nm) were stained with uranyl acetate and lead citrate and then visualized by TEM (H-600; Hitachi, Japan).

### Immunohistochemical (IHC) assay

Briefly, paraffin sections were deparaffinized in dimethylbenzene, rehydrated in a graded ethanol series, and incubated with the appropriate primary antibodies overnight at 4°C. Then, these sections were incubated with a secondary antibody for 30 min at 37°C. IHC staining intensity and the number of positive cells were evaluated by two independent pathologists.

### Immunofluorescence assay

Cells with the indicated treatments were cultured on coverslips. Then, the cells were fixed with 4% paraformaldehyde for 20 min and incubated with PBS containing 0.1% Triton X-100 for 5 min. The coverslips were shielded from light during the experiment. The coverslips were incubated with 100 nM rhodamine phalloidin (Cytoskeleton) for 45 min at room temperature for immunofluorescence analysis of the cytoskeleton. In addition, the coverslips were incubated with the anti-LC3 antibody (Cell Signaling Technology) overnight at 4°C for immunofluorescence staining of LC3. After being washed 3 times with PBS, the cells were incubated with the appropriate secondary antibody in 0.5% BSA at room temperature. 4',6-Diamidino-2-phenylindole (DAPI; Solarbio) was used for nuclear staining. Ultimately, the cells were visualized and imaged with confocal microscopy (FV10i; Olympus, Japan).

### Generation of xenografts

To confirm the effect of anlotinib-induced autophagy on the metastatic capacity of KHOS cells, six-week-old female BALB/c nude mice were purchased from Vital River (China), and then 3×10^6^ cells (KHOS, KHOS-shATG5 or KHOS-shNC) were intravenously injected into the tail veins of the mice (n=4 per group). After one week, the mice were intragastrically administered anlotinib at a dosage of 1 mg/kg daily for 30 days according to the group.

The mice were sacrificed upon termination of the study. The lungs of mice were collected for routine hematoxylin-eosin (HE) and IHC staining. Then, the number of metastatic nodules in the lungs was quantified. All the animal care and processes involved in this experiment were performed in conformity with the National Institutes of Health Guide for the Care and Use of Laboratory Animals. All animal experiments were approved by the Ethics Committees of The Affiliated Hospital of Qingdao University (China).

### Statistical analysis

SPSS v.21.0 software (IBM, USA) was used for statistical analyses. The data are reported as means±SD of at least three independent experiments. Statistical evaluation was carried out by one-way analysis of variance (ANOVA) and Student's *t*-test. A P value<0.05 was considered to be statistically significant.

## Results

### Anlotinib reduced the migration and invasion of human osteosarcoma cells

**Figure 1:**
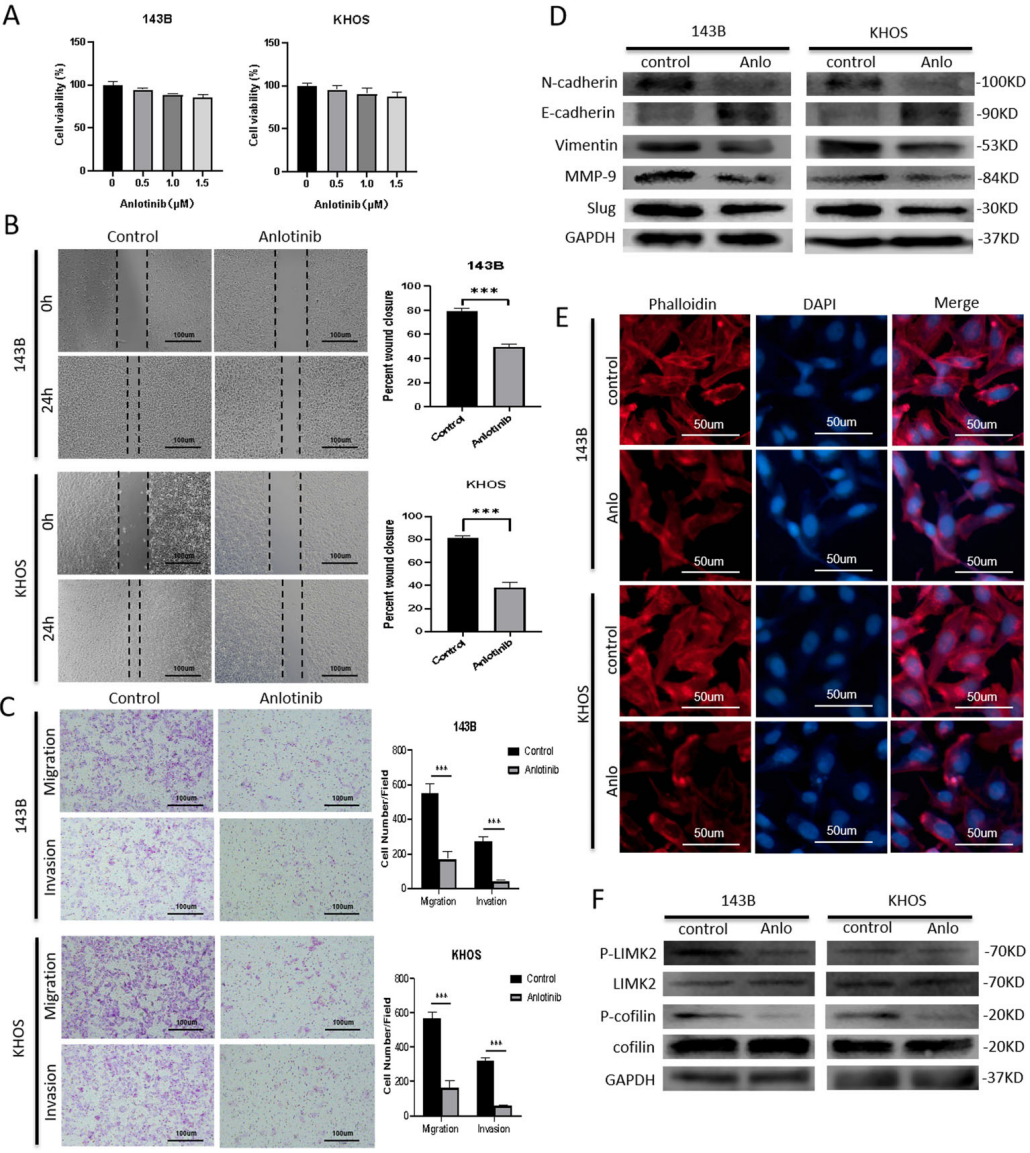
Anlotinib suppressed the migration and invasion of osteosarcoma cells. **A**, Anlotinib did not significantly influence the viability of 143B and KHOS cells at concentrations from 0.5 to 1.5 µM. **B** and **C**, Anlotinib treatment decreased the migration and invasion of 143B and KHOS cells, as determined by wound healing and transwell assays (scale bar 100 μm). **D**, Anlotinib treatment (Anlo) inhibited the epithelial-mesenchymal transition process, as shown by western blotting. **E**, More lamellipodial protrusions were formed in the control group than in the anlotinib treatment group. Representative images are shown, and nuclei were stained with DAPI. The scale bar represents 50 µm. **F**, Anlotinib treatment decreased the p-LIMK2 and p-cofilin levels, as shown by western blotting. All experiments were repeated three times. Data are reported as means±SD. ***P<0.001 (ANOVA and *t*-test).

Lung metastasis is an important factor affecting the prognosis of osteosarcoma patients (4), and how to control and treat pulmonary metastasis of osteosarcoma has been the focus of clinical practice. Thus, we investigated the inhibitory effects of anlotinib on the migration and invasion of human osteosarcoma cells using transwell and wound healing assays. First, we examined the effect of anlotinib on the viability of osteosarcoma cells by a CCK8 assay and found that anlotinib did not significantly influence the viability of 143B and KHOS cells at concentrations of 0.5, 1.0, and 1.5 µM (Figure 1A). The maximum concentration used was 1.5 µM for subsequent experiments in this study.

After anlotinib treatment, decreased migratory and invasive capacities of 143B and KHOS cells were observed in transwell and wound healing assays (Figure 1B and C).

### Anlotinib suppressed epithelial-mesenchymal transition and cytoskeletal rearrangement in osteosarcoma cells

Because both EMT and cytoskeletal rearrangement play a critical role in tumor metastasis (5), we first investigated the effects of anlotinib treatment on EMT-associated protein markers in 143B and KHOS cells using western blotting (WB). As shown in Figure 1D, anlotinib treatment significantly decreased the protein expression of mesenchymal markers, including Vimentin and N-cadherin. However, the expression of the epithelial marker E-cadherin was increased after anlotinib treatment. Additionally, the expression of matrix metalloproteinase-9 (MMP-9) and the transcription factor Slug were decreased after anlotinib treatment (Figure 1D).

An immunofluorescence assay of the cytoskeleton in 143B and KHOS cells was conducted using confocal microscopy. After anlotinib treatment, F-actin was arranged mostly in the cytoplasm of the anlotinib treatment group, while obvious lamellipodial protrusions and colored F-actin filaments were visualized in the cell margin in the control group (Figure 1E). Since LIMK and cofilin are critical regulators of cytoskeletal rearrangement (6), we evaluated changes in LIMK and cofilin activation with anlotinib treatment. Decreased phosphorylation-mediated activation of cofilin and LIMK2 was observed in the anlotinib treatment group compared to the control group using WB (Figure 1F).

Collectively, these findings strongly indicate that anlotinib significantly inhibited the migration and invasion of osteosarcoma cells by regulating EMT and cytoskeletal rearrangement.

### Anlotinib induced human osteosarcoma cell autophagy through ATG5

**Figure 2:**
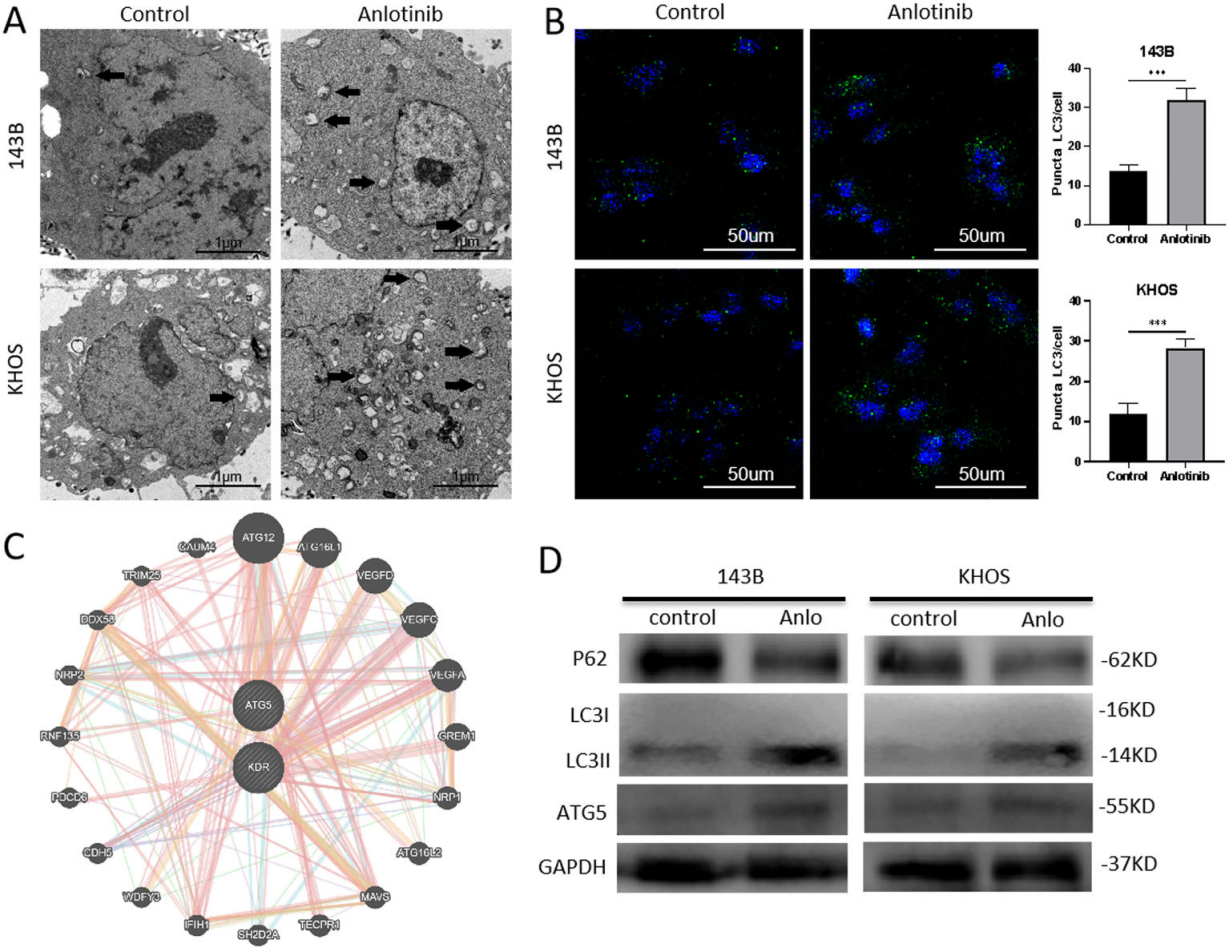
Anlotinib induced human osteosarcoma cell autophagy through ATG5. **A**, Representative transmission electron microscopy images revealed ultrastructural differences during autophagy with anlotinib treatment. The arrows indicate the greater number of autophagic vacuoles in the anlotinib treatment group (scale bar 1 μm). **B**, Higher fluorescence from formed LC3-II puncta was observed in the anlotinib treatment group (scale bar 50 μm). Data are reported as as means±SD. ***P<0.001 (*t*-test). **C**, Bioinformatics analysis for the interaction between KDR and ATG5 (http://genemania.org/). **D**, Autophagy-related proteins, including ATG5, were evaluated by western blotting.

Previous studies have confirmed that anlotinib can induce autophagy in a variety of tumors, such as lung cancer, breast cancer, glioblastoma, and colon cancer (7-9). To the best of our knowledge, there are no studies on anlotinib-mediated autophagy in osteosarcoma. To determine the effect of anlotinib on autophagy in osteosarcoma, TEM was used to detect ultrastructural changes upon anlotinib treatment. Osteosarcoma cells treated with anlotinib exhibited a higher number of autophagic vacuoles than control cells (Figure 2A). As shown in Figure 2B, osteosarcoma cells treated with anlotinib exhibited higher fluorescence from formed LC3-II puncta, indicating an increase in LC3-II in autophagosomes. ATG5, a key gene in the autophagy process, may be involved in anlotinib-induced autophagy, as predicted through bioinformatics analysis (Figure 2C). Subsequently, autophagy-related proteins were investigated by WB. Increased LC3-II expression and decreased p62 expression were detected in anlotinib-treated 143B and KHOS cells (Figure 2D). In addition, elevated ATG5 expression was detected in anlotinib-treated 143B and KHOS cells (Figure 2D). In summary, our results suggested that anlotinib induced autophagy in osteosarcoma cells.

### Inhibition of anlotinib-induced autophagy enhanced the inhibitory effects of anlotinib on osteosarcoma metastasis by regulating EMT and cytoskeletal rearrangement through ATG5

Anlotinib has been proven to have antitumor effects in a variety of solid tumors, and it also plays an important role in the treatment of osteosarcoma and soft tissue sarcoma (10). However, the specific mechanism of action of anlotinib in osteosarcoma is still unclear, especially the effect and mechanism of the autophagy induced by anlotinib. As a double-edged sword in tumor cells, autophagy may either inhibit or promote migration and invasion, depending on the tumor cell context (11). In some metastatic tumors, robust autophagy is associated with invasiveness and poor survival (12-15). Thus, the regulation of autophagy may enhance the curative effect of drug therapy.

**Figure 3:**
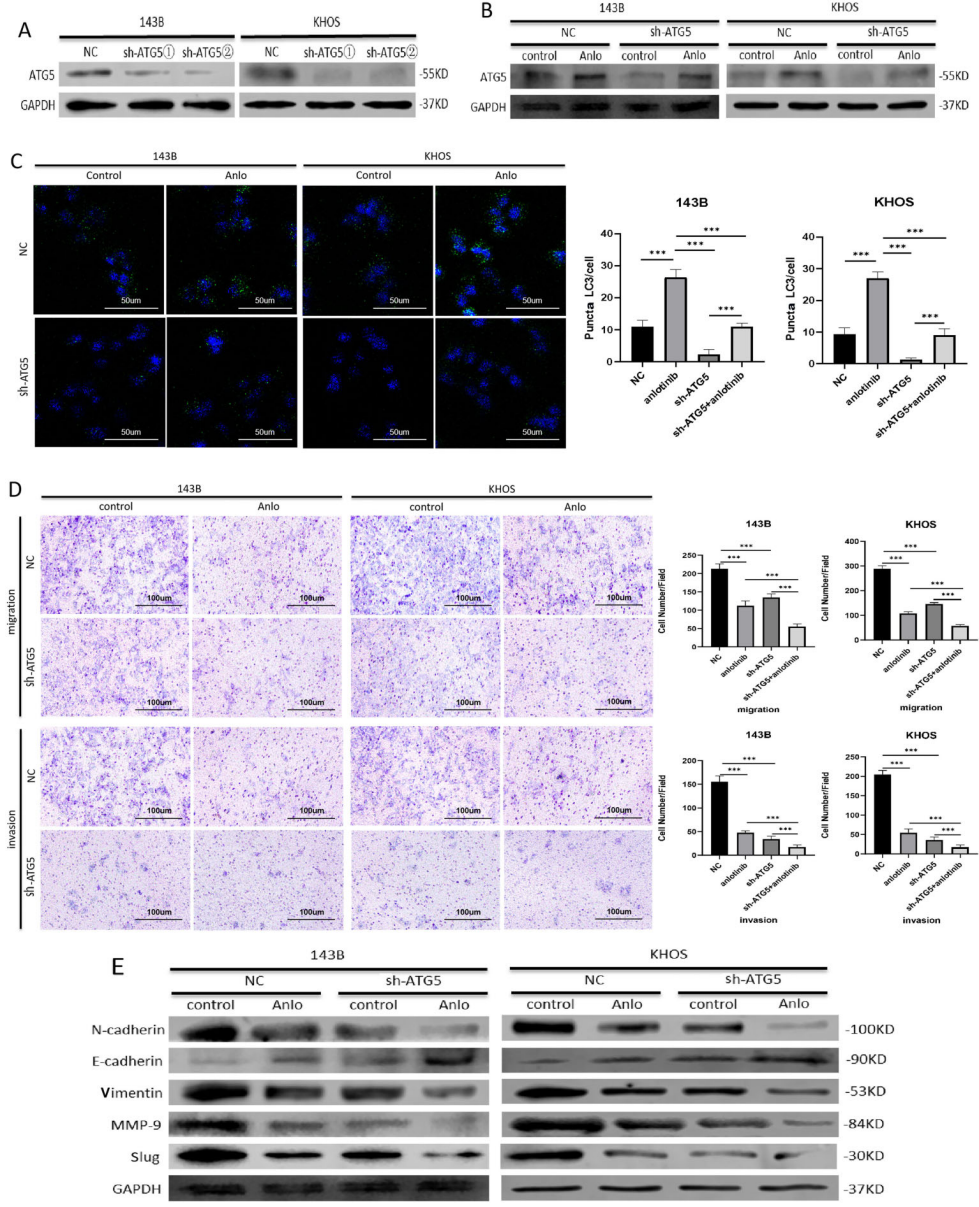
Inhibition of anlotinib-induced autophagy enhanced the inhibitory effects of anlotinib (Anlo) on osteosarcoma metastasis by regulating epithelial-mesenchymal transition (EMT) through ATG5. **A**, 143B and KHOS cells were transfected with short hairpin (sh) RNA sequences targeting ATG5, and the knockdown efficiency was evaluated by western blotting. **B** and **C**, ATG5 knockdown obviously suppressed anlotinib treatment-induced autophagic flux, as shown by western blot and immunofluorescence analyses (scale bar 50 μm). **D**, ATG5 silencing significantly enhanced the inhibitory effects of anlotinib on the invasion and migration of 143B and KHOS cells, as determined by transwell assays (scale bar 100 μm). **E**, Anlotinib treatment inhibited the EMT process, which was enhanced by ATG5 silencing. All experiments were repeated three times. Data are reported as means±SD. ***P<0.001 (ANOVA).

**Figure 4:**
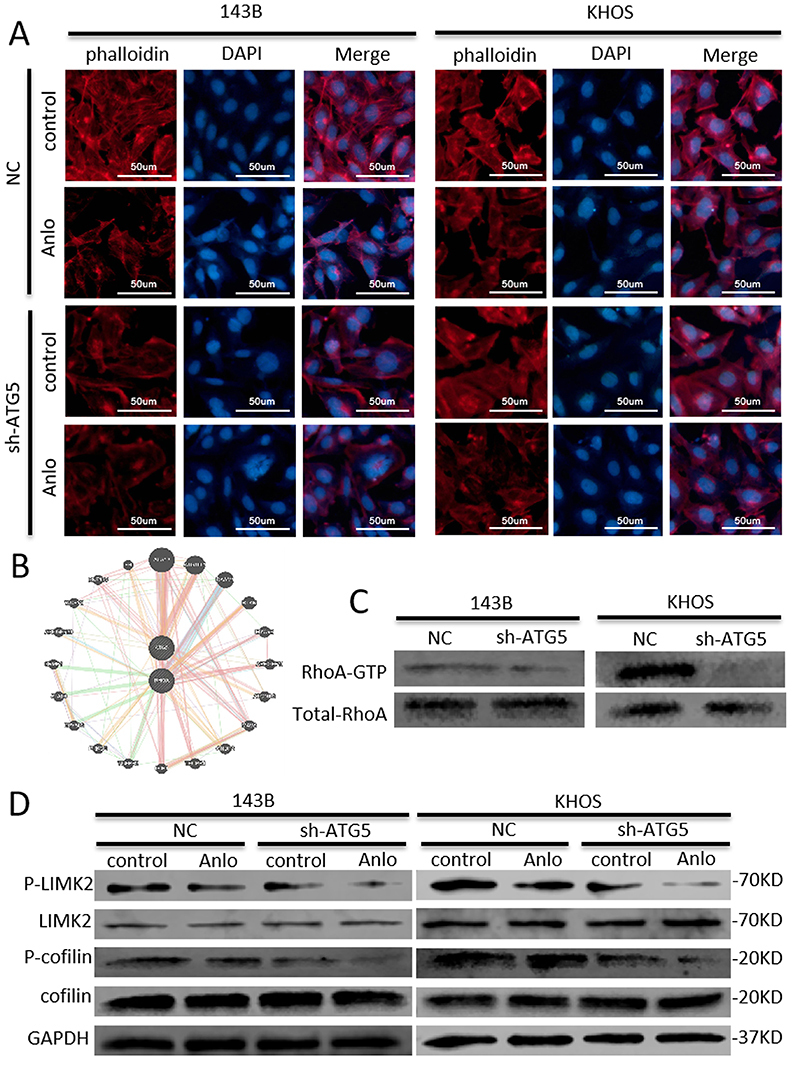
Inhibition of anlotinib-induced autophagy enhanced the inhibitory effects of anlotinib (Anlo) on osteosarcoma metastasis by regulating cytoskeletal rearrangement through ATG5. **A**, Anlotinib treatment influenced cytoskeletal rearrangement, which was enhanced by ATG5 silencing (scale bar 50 μm). **B**, Bioinformatics prediction of the interaction between ATG5 and RhoA (http://genemania.org/). **C**, RhoA activation was decreased after ATG5 depletion in 143B and KHOS cells, as determined using the GTPase activity assay. **D**, ATG5 knockdown enhanced the anlotinib-induced decline in p-LIMK2 and p-cofilin levels, as determined by western blotting.

To confirm the role of anlotinib-induced autophagy in osteosarcoma cell metastasis, we knocked down ATG5 with short hairpin RNA (shRNA) to inhibit autophagy (Figure 3A). ATG5 knockdown obviously suppressed anlotinib treatment-induced autophagic flux, as shown by WB (Figure 3B). Similar results were found in the immunofluorescence assay for LC3-II (Figure 3C). The transwell assay results revealed that autophagy inhibition mediated by ATG5 silencing significantly enhanced the inhibitory effects of anlotinib on the migration and invasion of 143B and KHOS cells (Figure 3D). As a key player in autophagy (16), ATG5 has been demonstrated to participate in the regulation of EMT (17). Mechanistically, anlotinib treatment inhibited the EMT process and influenced cytoskeletal rearrangement, while both EMT and cytoskeletal rearrangement were enhanced by ATG5 silencing (Figure 3E and 4A). Additionally, there may be an interaction between ATG5 and RhoA, as predicted through bioinformatics analysis (Figure 4B). According to the results, RhoA activation was decreased in both the shATG5-KHOS and shATG5-143B groups compared with the control groups in the GTPase activity assay (Figure 4C). As critical downstream molecules of RhoA, the levels of p-cofilin and p-LIMK2 were also investigated (18). As shown in Figure 4D, ATG5 knockdown strengthened the anlotinib-induced decline in p-LIMK2 and p-cofilin levels in 143B and KHOS cells.

Taken together, the above results obtained through genetic inhibition of autophagy revealed that anlotinib-induced protective autophagy promoted the invasion and migration of human osteosarcoma cells by regulating EMT and cytoskeletal rearrangement through ATG5. The inhibitory effect of anlotinib on metastasis can be enhanced by inhibiting autophagy.

### Autophagy maintained the migration and invasion of osteosarcoma cells through EMT and the RhoA-ROCK-LIMK2 pathway

**Figure 5:**
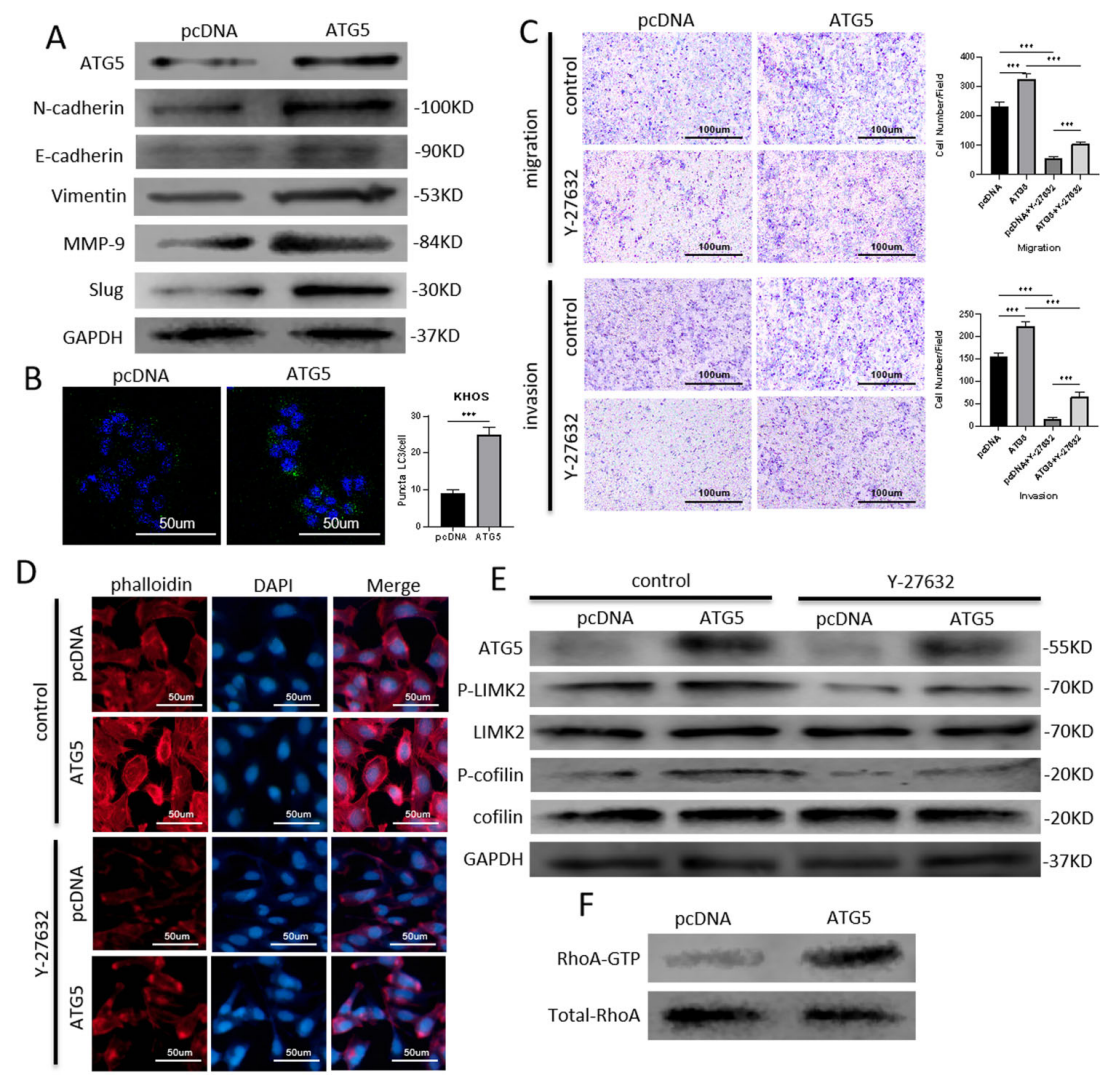
Autophagy maintained the migration and invasion of osteosarcoma cells through EMT and the RhoA-ROCK-LIMK2 pathway. **A**, ATG5 overexpression increased MMP-9 expression and enhanced the EMT process, as shown by western blotting. **B**, Immunofluorescence staining of LC3-II indicated the autophagic flux induced by ATG5 overexpression (scale bar 50 μm). **C**, Y-27632 decreased the migration and invasion of osteosarcoma cells, as determined by transwell assays, and these effects were reversed by ATG5 overexpression (scale bar 100 μm). **D**, Y-27632 inhibited cytoskeletal rearrangement, which was also reversed by ATG5 overexpression (scale bar 50 μm). **E**, The Y-27632-induced decline in the p-LIMK2 and p-cofilin levels was reversed by ATG5 overexpression. **F**, ATG5 overexpression also resulted in increased RhoA activation. Data are reported as means±SD. ***P<0.001 (ANOVA and *t*-test).

Furthermore, to validate the effect of anlotinib-induced autophagy on osteosarcoma cell metastasis, an ATG5 overexpression vector was transfected into KHOS cells. Then, increased MMP-9 expression and elevated EMT were detected by WB after ATG5 overexpression (Figure 5A). Additionally, ATG5 overexpression induced autophagic flux, as determined by immunofluorescence staining of LC3-II (Figure 5B). Y-27632 decreased the migration and invasion of osteosarcoma cells, while these effects were reversed by ATG5 overexpression (Figure 5C). Mechanistically, Y-27632 inhibited cytoskeletal rearrangement, and this effect was also reversed by ATG5 overexpression (Figure 5D). The Y-27632-induced declines in p-LIMK2 and p-cofilin were reversed by ATG5 overexpression (Figure 5E). ATG5 overexpression also resulted in increased RhoA activation (Figure 5F).

Ultimately, these findings, obtained through genetic enhancement of autophagy combined with treatment with a cytoskeleton signaling pathway inhibitor, indicated that anlotinib-induced autophagy promoted the metastasis of human osteosarcoma cells via RhoA-ROCK-LIMK2 signaling and EMT.

### Inhibition of ATG5-dependent autophagy induced by anlotinib enhanced the inhibitory effects of anlotinib on osteosarcoma pulmonary metastasis *in vivo*

**Figure 6:**
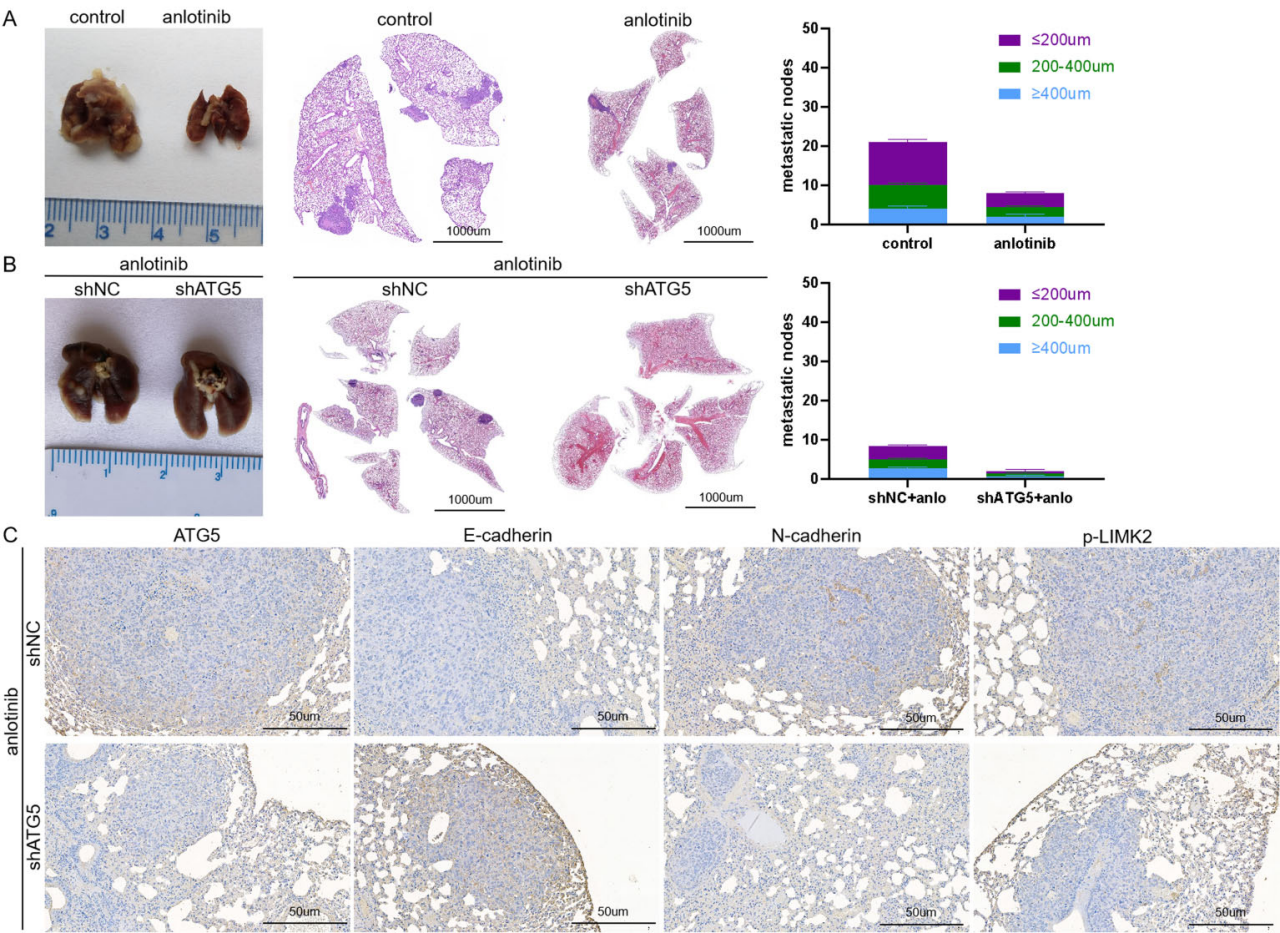
Inhibition of ATG5-dependent autophagy enhanced the inhibitory effects of anlotinib on osteosarcoma pulmonary metastasis *in vivo*. **A** and **B**, Representative images of lungs from the corresponding groups are shown, and pulmonary metastases were quantified. Fewer pulmonary metastases were observed in the anlotinib treatment group and shATG5 group than in the corresponding control groups (scale bar 1000 μm). **C**, Immunohistochemical analyses of E-cadherin, N-cadherin, ATG5, and p-LIMK2. Representative images are shown at 200× magnification (scale bar 50 μm). Data are reported as means±SD (n=4).

To further verify the effects of ATG5-dependent autophagy induced by anlotinib on the metastatic ability of KHOS cells, an *in vivo* xenograft model was built in accordance with the tumor xenograft model. The anlotinib treatment group exhibited significantly fewer lung metastatic nodules than the control group (Figure 6A). Moreover, in mice with concomitant anlotinib treatment, fewer pulmonary metastases were observed in the shATG5 group than in the shNC group; representative HE staining images are shown in Figure 6B. Furthermore, IHC staining revealed elevated E-cadherin membranous expression in pulmonary metastases of the shATG5 group. Moreover, the protein levels of N-cadherin, ATG5, and p-LIMK2 were reduced in pulmonary metastases of the shATG5 group compared with the shNC group (Figure 6C).

Taken together, these data indicated that anlotinib treatment induced ATG5-dependent autophagy and that inhibition of autophagy enhanced the inhibitory effects of anlotinib on osteosarcoma metastasis through EMT and the RhoA-ROCK-LIMK2 pathway.

## Discussion

In recent years, targeted antiangiogenic drugs have achieved good therapeutic effects in a variety of cancers, including thyroid cancer, colon adenocarcinoma, non-small cell lung cancer, renal clear cell cancer, and soft tissue sarcoma (19-23).

In a limited number of previous studies, it was confirmed that anlotinib suppressed osteosarcoma growth and metastasis via dual blockade of VEGFR2 and MET (10), and anlotinib was also found to reverse multidrug resistance (MDR) in osteosarcoma by inhibiting p-glycoprotein (PGP1) function (24). Some studies have also confirmed that certain factors play an important role in the treatment of osteosarcoma. For example, miR-596 suppresses the expression of survivin and enhances the sensitivity of osteosarcoma cells to the targeted molecular agent anlotinib (25), and treatment with a PI3K inhibitor was found to impair tumor progression and enhance sensitivity to anlotinib in anlotinib-resistant osteosarcoma (26). The role of autophagy in tumor metastasis is still controversial, and the underlying mechanisms remain unclear. Little is known about the effects of anlotinib on metastasis, especially the relationship between autophagy and metastasis in human cancer cells.

Previous studies have confirmed the role of anlotinib-induced autophagy in several cancers, including non-small cell lung cancer, breast cancer, glioma, and colon cancer (27). However, these studies mainly focused on the relationships between anlotinib-induced autophagy and tumor cell proliferation and apoptosis.

In the present study, we found that a low concentration of anlotinib, which did not significantly affect cell viability, attenuated metastasis and induced autophagy in osteosarcoma. Because the regulation of autophagy may influence the curative effect of anlotinib treatment, we further investigated the possible effects of anlotinib-induced autophagy on the metastasis of osteosarcoma cells and explored the underlying mechanisms. Interestingly, as shown in the results, inhibition of anlotinib-induced autophagy by ATG5 knockdown further enhanced the inhibitory effect of anlotinib on the invasion and migration of osteosarcoma cells. In contrast, inducing autophagy via ATG5 overexpression in osteosarcoma cells increased their invasion and migration abilities. These results suggested that anlotinib induced protective autophagy by activating ATG5, which promoted osteosarcoma cell metastasis.

Mechanistically, activation of EMT plays an important role in the metastasis of tumor cells, and some epithelial or mesenchymal markers and transcription factors are differentially expressed during this process (28). Previous studies also revealed the dual effect of autophagy on EMT regulation (29). In our study, we found that anlotinib treatment decreased the expression of mesenchymal markers and increased that of epithelial markers. Furthermore, these effects were enhanced by ATG5 depletion, which resulted in reduced metastasis of osteosarcoma cells. ATG5 overexpression promoted EMT as well as osteosarcoma cell migration. All of these results indicated that anlotinib-induced autophagy accelerated EMT through ATG5.

The cytoskeletal structure also plays a key role in cancer cell motility and invasiveness, which are sophisticated processes that involve cytoskeletal rearrangement, lamellipodia formation, and membrane ruffling (30). Both cofilin and LIMK play an important role in the regulation of actin reorganization, which is also regulated by RhoA activation (31). Previous studies have shown that autophagy plays a multifaceted role in the regulation of the cytoskeleton (32,33). Herein, we found that ATG5 silencing decreased RhoA activation, while ATG5 overexpression increased RhoA activation. Moreover, the reduced formation of lamellipodial protrusions after anlotinib treatment was enhanced by ATG5 knockdown. The increase in lamellipodial protrusions induced by ATG5 overexpression was reversed by Y-27632, an inhibitor of cytoskeletal pathways. These results suggested that anlotinib-induced autophagy could significantly affect cytoskeletal rearrangement in osteosarcoma cells.

**Figure 7:**
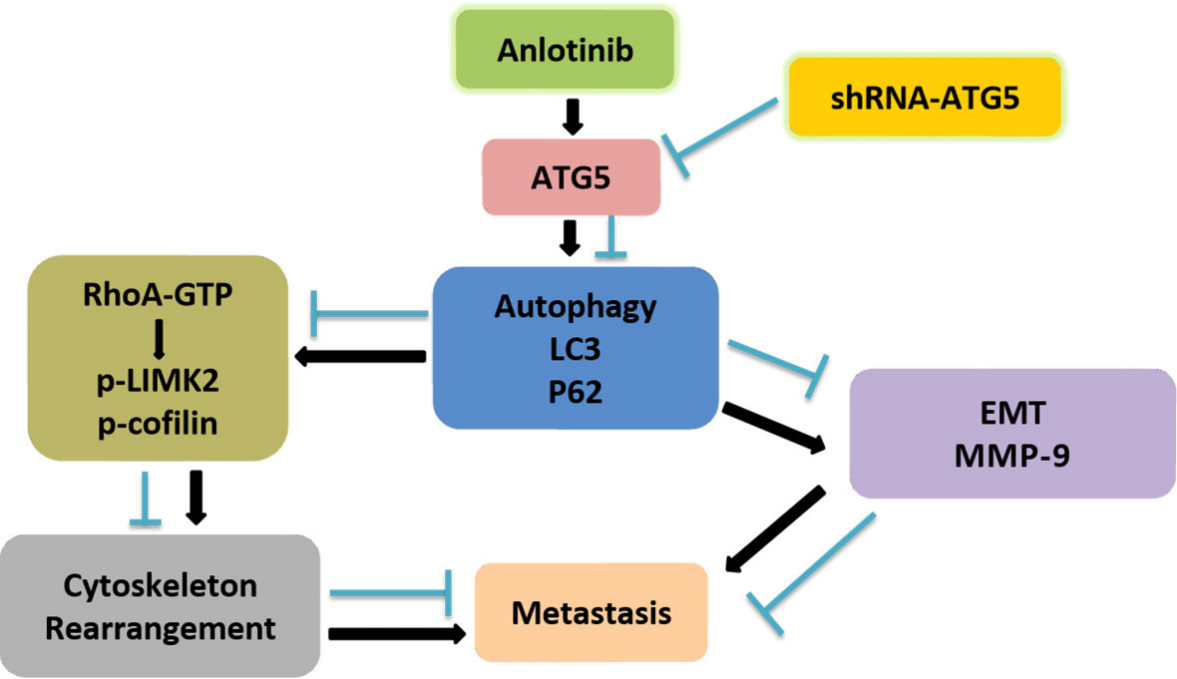
A schematic showing the regulatory mechanism of anlotinib-induced autophagy in osteosarcoma metastasis. EMT: epithelial-mesenchymal transition.

In summary, research on the correlation between anlotinib-induced autophagy and metastasis is scarce. In this study, we demonstrated that anlotinib can induce protective autophagy in osteosarcoma cells and that the autophagy induced by anlotinib can facilitate EMT and regulate cytoskeletal rearrangement through ATG5, through which anlotinib-induced autophagy promotes the metastasis of human osteosarcoma cells (Figure 7).

Indeed, our study mainly confirmed the role and mechanism of autophagy induced by anlotinib on metastasis of osteosarcoma. However, since anlotinib is mostly a multi-target tyrosinase inhibitor, how it affects the expression of autophagy-related proteins such as ATG5 to play its role still needs further research and exploration. The role of anlotinib-induced autophagy in the proliferation of osteosarcoma, especially in pyroptosis, is also very worthy of our attention and should be studied further.

### Conclusion

Our study demonstrated for the first time the prometastatic role of anlotinib-induced autophagy in osteosarcoma. Anlotinib-induced autophagy promoted migration and invasion by activating EMT and cytoskeletal rearrangement through ATG5 both *in vitro* and *in vivo*, and inhibition of anlotinib-induced autophagy enhanced the inhibitory effects of anlotinib on osteosarcoma metastasis. Thus, the therapeutic effect of anlotinib can be improved by combination treatment with autophagy inhibitors, which provides a new direction for the treatment of metastatic osteosarcoma.
